# Fundamentals of Oncologic PET-CT Reporting

**DOI:** 10.4274/Mirt.46320

**Published:** 2013-04-05

**Authors:** Bijan Bijan, Giselle Melendres, Tuan Nguyen

**Affiliations:** 1 University of California Davis Medical Center, Nuclear Medicine and Radiology, USA; 2 University of California Davis Medical Center, Associate Professor of Radiology, USA; 3 Sutter Medical Group, Sutter Roseville Medical Center, Diagnostic Medical Imaging Division, USA

**Keywords:** positron-emission tomography/computed tomography, nuclear medicine

Over the past 15 years, from the inception of hybrid imaging and introduction of PET-CT to the armamentarium of medical diagnostic departments, numerous styles of reporting have been employed. The type of reporting is mostly derived by the organizational structure of the imaging department. Institutions with separate Radiology and Nuclear Medicine departments usually generate two separate reports. The PET portion of the interpretation is made in the light of anatomical landmarks provided by the CT portion of the study. A separate CT report is also generated. In many instances, a third report is created fusing the separate PET and CT reports. The downsides to this system are the inefficiency of interpretation and the potential confusion for referring physicians when the separate PET and CT reports don’t completely match. Needless to say, CT also provides much more information than simple anatomical landmarks, many of which may be crucial to the management of the patient.

The more appropriate method of interpretation is for the PET and CT portions of the examination to be interpreted by a dual trained and credentialed imager, creating a single, comprehensive report encompassing both PET and CT findings in the body of the report. In the impression of the report, both sets of findings are correlated and appropriate conclusions and recommendations are made. There are two main downsides to this “perfect” reporting system. Dual trained imagers are not that abundant in the imaging community and healthcare payers may be more likely to decrease the payment to a single reimbursement for the entire examination.

The following is the authors’ recommendation of reporting a PET-CT study:

1. Patient demographics including name, last name, date of birth, medical record number, inpatient / outpatient status, study-accession number.

2. Study related data including date of service, time of service, location of service

**3. Comparison:**

Any related comparative studies including prior PET-CT, CT, Ultrasound, MR, etc. Preferably with mention of date and technique (with contrast or without oral and/or IV contrast) 

**4. Indication:**

Common indications include initial staging, response to treatment and assessmentfor recurrence. Indications such as detection of the primary focus of cancer of unknown origin haven’t been widely used yet.

**5. History:**

Brief relevant history, including the histological subtype of malignancy, if known, any applied treatment (surgery, radiation, chemotherapy, etc) with documentation of time and duration, and any relevant tumor markers. Recently, in California, a new regulation has created some confusion as to what can be legally written in patient’s report as part of history. Based on the new California regulation, any “tissue-histological diagnosis” or laboratory values like hepatitis titer and HIV antibody status may not be written in the radiology report to protect patient privacy in released electronic medical record, i.e. the radiology report. This regulation may not exist in other states yet!

**6. Technique:**

a. Any specific patient preparation given to patient like the duration of NPO and if any high-protein diet was used

b. Documentation of blood glucose level

c. The employed CT technique including acquisition parameters and image reconstruction algorithms.

d. If any IV or Oral contrast is utilized. On occasion rectal water is given.

e. The PET technique, including the administered radioisotope dose, route, site and time of administration, timing of data acquisition with respect to dose administration. In many institutions the name of the individual who administered the dose is also documented on the report (for medico-legal purposes).

f. Any complication or technical difficulties or limitations, including extravasation, patient motion, contamination, etc

**7. Radiation dose documentation:**

a. California law mandates the radiation-dose reporting for every CT study. b. Radiation received from FDG may also be documented.

**8. Quality of the study:**

A quick review of cine images may reveal any technical issues including motion, metallic objects or any other sorts of artifact. Unusual muscle activity may also affect the accuracy of your interpretation.

**9. Findings:**

a. PET report: (Editor’s method) Cine images are initially reviewed. Then trans-axial images and coronal images are reviewed. A checklist is generated of all of the detected positive 

PRECIST/RECIST guidelines are used to facilitate communication with oncology colleagues. Proper measurement of SUV, SUVmax and average SUV is imperative. Also review of prior studies is needed so that the measurements are comparable.

b. CT report: The CT is reviewed in detail in the same systematic way a radiologist reviews a CT-only study. All relevant and incidental abnormalities are listed.

**10. Conclusion/Impression:**

a. The abnormal foci from PET are matched to CT abnormalities and an interpretation is rendered. Example: Although the size of the aortocaval node shows no appreciable morphological change since the prior study, the degree of its metabolic activity is decreased. This may signify favorable response to therapy.

b. Several PET findings may be physiological in nature. These findings usually are not included in the impression. The main goal of the report-impression is to be concise and precise.

c. Certain PET findings are not directly related to current oncologic condition of the patient but deserve medical attention, including diffuse thyroidal uptake. Therefore an appropriate recommendation should be given.

d. CT findings with clinical significance should be explicitly reported in the impression with appropriate recommendations. Example: Abdominal aortic aneurysm, coronary arterial calcification, pleural and pericardial effusion, hernias, renal stones, etc.

**11. Staging/Re-staging:**

a. Oncologic PET-CT reports may contain imaging staging of the disease. The editors prefer using TNM staging, unless requested otherwise by the referring oncologists. RECIST guideline modified for PET, so-called PERCIST, is a way to standardize the PET-CT reports and is highly encouraged by the editors. Unifying the staging guidelines of PET and CT is crucial.

b. Comparison with prior studies may pose various challenges. Many of the PET-CT findings cannot be precisely correlated with other modalities including MRI and ultrasound. Vice versa, very small lesions, detected by MR and Ultrasound may be too small for PET to detect. In these contexts, the imager needs to use her/his experience and Gestalt impression to make appropriate follow-up/work-up 

